# Quantifying Radiation Effects of Iodinated Contrast Media in Pediatric CT Using DNA Damage Biomarkers

**DOI:** 10.3390/ijms27146145

**Published:** 2026-07-09

**Authors:** Otilia Nuta, Ainur Kenessova, Antonio Sarno, Dinara Jumadilova, Tairkhan Dautov

**Affiliations:** 1 Department of Biology, School of Sciences and Humanities, Nazarbayev University, Astana 010000, Kazakhstan; 2 Department of Medicine, School of Medicine, Nazarbayev University, Astana 010000, Kazakhstan; ainur.kenessova@nu.edu.kz (A.K.); dinara.jumadilova@nu.edu.kz (D.J.); 3 Department of Physics ‘Aldo Pontremoli’ and INFN Milano, Università degli Studi di Milano, 20133 Milan, Italy; antonio.sarno@unimi.it; 4 Clinical and Academic Department of Radiology and Nuclear Medicine, Heart Center, Corporate Fund “University Medical Center”, Astana 010000, Kazakhstan; 5 Clinical and Academic Department of Radiology and Nuclear Medicine, Corporate Fund “University Medical Center”, Astana 010000, Kazakhstan; tairkhan.dautov@gmail.com

**Keywords:** computed tomography, pediatric imaging, iodinated contrast media, DNA damage, γ-H2AX, radiation dosimetry

## Abstract

This prospective single-center study investigated the impact of iodinated contrast agents (Ultravist/iopromide and Visipaque/iodixanol) on DNA double-strand breaks during pediatric chest computed tomography (CT). Twenty-six children underwent medically indicated chest CT between March 2022 and November 2024: unenhanced (*n* = 7), Ultravist-enhanced (*n* = 10), and Visipaque-enhanced (*n* = 9). Blood samples were collected before and 30 min post-CT. DNA damage was quantified by analyzing γ-H2AX and 53BP1 foci formation in peripheral lymphocytes using immunofluorescence microscopy. All groups showed significant increases in DNA damage following CT. The absolute increases were 0.915 ± 0.250 foci/cell (unenhanced), 0.972 ± 0.279 (Ultravist), and 1.261 ± 0.251 (Visipaque), representing 6.2% and 37.8% higher values in contrast groups. However, these differences between groups were not statistically significant (*p* = 0.579, Kruskal–Wallis test), with small effect sizes (Cohen’s d < 0.5 for all comparisons). Contrast-enhanced protocols employed higher radiation doses than unenhanced scans (CTDIvol: 4.04–4.57 mGy vs. 2.66 mGy). When DNA damage was normalized for CTDIvol, no significant differences were observed between groups (*p* = 0.659). DNA damage showed no significant correlation with dosimetric parameters. When accounting for radiation dose differences, iodinated contrast agents do not enhance radiation-induced DNA double-strand breaks in pediatric chest CT, confirming the safety of contrast-enhanced CT.

## 1. Introduction

Computed tomography (CT) represents a fundamental imaging modality in pediatric diagnostics, offering superior spatial resolution and exceptional tissue differentiation capabilities. CT utilization in pediatric populations has exhibited a marked upward trend, with an estimated annual increase of 8–10% over the past two decades [1,2,3]. This trend has raised concerns regarding stochastic radiation effects, particularly in pediatric cohorts where intrinsic radiosensitivity is increased, resulting in a 2–3-fold greater lifetime attributable risk compared to adults [4,5,6,7].

Iodinated contrast media (ICM) significantly augment diagnostic yield in CT examinations through enhancement of vascular structures and alteration of tissue attenuation characteristics. The pharmacokinetic profiles of non-ionic monomeric (e.g., Ultravist/iopromide) and non-ionic dimeric (e.g., Visipaque/iodixanol) agents differ substantially in terms of osmolality, viscosity, and iodine delivery efficiency [8]. Theoretical concerns have been raised that these high-atomic-number (Z = 53) agents might potentiate radiation-induced DNA damage through (1) photoelectric effect amplification with resultant secondary electron production, (2) augmented free radical generation, and (3) direct chemical genotoxicity [8,9,10]. However, clinical evidence remains contradictory, with some studies reporting enhancement effects while others, when accounting for dose differences, find no biological amplification [11]. Recent Monte Carlo studies have confirmed that contrast agents can increase organ radiation dose by approximately 30% due to photoelectric absorption enhancement [12].

DNA double-strand breaks (DSBs) represent the most cytotoxic lesions induced by ionizing radiation, with profound implications for genomic integrity and cellular viability [13]. The phosphorylation of histone variant H2AX at serine 139 (γ-H2AX) occurs within minutes of DSB formation via ATM/ATR/DNA-PK-mediated phosphorylation cascades, creating a megabase-scale chromatin domain that facilitates recruitment of repair factors [14,15]. The p53-binding protein 1 (53BP1) subsequently localizes to these domains, functioning as a critical regulator of DSB repair pathway choice between homologous recombination and non-homologous end joining [16]. The temporal and spatial co-localization of γ-H2AX and 53BP1 foci provides an exquisitely sensitive method for quantitative assessment of DSBs, with detection thresholds approximating 1 mGy in peripheral lymphocytes [13,17].

In vitro and ex vivo investigations have demonstrated dose-enhancement ratios of 1.3–2.0 for various ICM concentrations in adult peripheral blood lymphocytes exposed to diagnostic X-ray energies [8,18]. However, extrapolation of these findings to pediatric patients is confounded by differences in radiation dose optimization protocols, anatomical dimensions, and potentially divergent DNA damage response kinetics [19]. The scarcity of pediatric-specific data represents a significant gap in the scientific literature, particularly given the heightened radiation vulnerability in this demographic. While studies have examined DNA damage in pediatric patients during cardiac catheterization [20] and diagnostic CT examinations [21], data comparing contrast-enhanced versus unenhanced protocols in children remain limited.

Despite conflicting evidence, no pediatric study has systematically compared DNA damage between contrast-enhanced and unenhanced CT while accounting for the fundamental dose confounding inherent in clinical protocols. This investigation aims to determine whether iodinated contrast agents (non-ionic monomeric Ultravist/iopromide and non-ionic dimeric Visipaque/iodixanol) enhance radiation-induced DSB formation during pediatric chest CT examinations beyond what would be expected from radiation dose alone. Through quantitative assessment of γ-H2AX/53BP1 co-localized foci in patient-derived lymphocytes combined with patient-specific dosimetry, we examine whether apparent DNA damage increases reflect contrast-mediated biological radiosensitization or the higher radiation doses inherently required for contrast-enhanced protocols in clinical practice. This dose-normalized approach provides the first pediatric clinical evidence examining DNA damage in contrast-enhanced CT, contributing important data to the ongoing debate in the radiological literature.

## 2. Results

### 2.1. Patient Characteristics

Table 1 reports patient demographics and baseline characteristics.

The median age was 10.5 months (range 1.8–123 months). The median weight was 7.5 kg (range 4.2–28.0 kg) and the median height was 74 cm (range 55–144 cm).

Patient characteristics by group and radiation dose parameters are shown in Table 2.

Baseline demographics were generally comparable across the three groups, though female patients were older than male patients across all groups, with mean ages ranging from 31.4 to 65.5 months compared to 11.4 to 19.0 months for males.

### 2.2. Scanner Reported and Calculated Dose Metrics

Radiation dose parameters differed substantially between groups, reflecting the different clinical protocols used for contrast-enhanced versus unenhanced examinations. CTDIvol values were lowest in the unenhanced group (2.66 ± 0.42 mGy) and higher in contrast-enhanced groups: Ultravist (4.04 ± 0.31 mGy) and Visipaque (4.57 ± 0.76 mGy), representing 52% and 72% increases respectively compared to unenhanced scans.

DLP values showed a different pattern, with the Visipaque group having the lowest values (25.9 ± 4.7 mGy·cm) despite the highest CTDIvol, reflecting substantially shorter calculated scan lengths (6.78 ± 1.12 cm) compared to unenhanced (14.02 ± 2.54 cm) and Ultravist (9.58 ± 0.88 cm) protocols. Using NCICT age-specific dosimetry calculations [22], effective doses ranged from 0.99 to 3.68 mSv across the patient cohort, with corresponding lung doses of 2.31 to 9.16 mGy and estimated blood doses of 1.07 to 4.27 mGy.

NCICT-calculated organ doses are presented in Table 3. Mean lung doses were 4.66 ± 0.91 mGy for unenhanced, 5.37 ± 0.69 mGy for Ultravist, and 4.17 ± 0.5 mGy for Visipaque groups. Heart wall and active bone marrow doses showed similar patterns across groups (Table 3).

**Figure 1:**
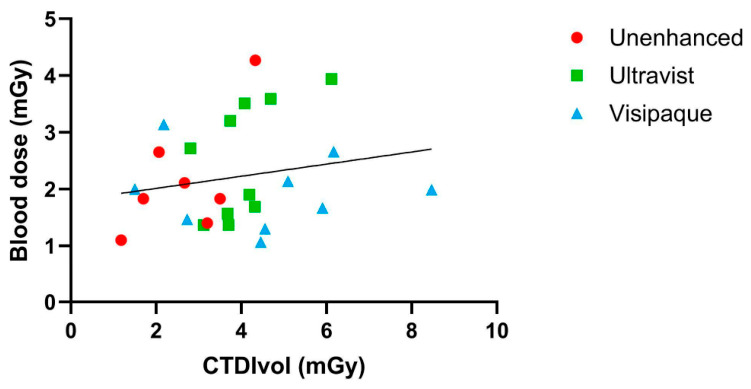
Relationship between NCICT-derived blood dose and scanner-reported CTDIvol. Each point represents one patient: unenhanced (red circles), Ultravist (green squares), and Visipaque (blue triangles). Solid lines represent linear regression fits for each group.

Figure 1 reports the blood dose as function of the scanner-reported CTDIvol. The negligible correlation (r^2^ = 0.06) reflects the patient-specific nature of Monte Carlo organ dose calculations which account for individual anatomical variations (age, body size, tissue composition) not captured by scanner-reported dose metrics.

### 2.3. γ-H2AX Focus Yield After CT

Before undergoing CT scans, all three groups had similar baseline levels of γ-H2AX foci per cell. The unenhanced group had 3.65 ± 0.39 foci per cell, compared to 3.71 ± 0.28 foci per cell in the Ultravist-enhanced group, and 3.82 ± 0.42 foci per cell in the Visipaque-enhanced group. There was no significant difference observed between the groups (*p* > 0.05), confirming comparable baseline DNA damage levels across all patient groups.

**Figure 2:**
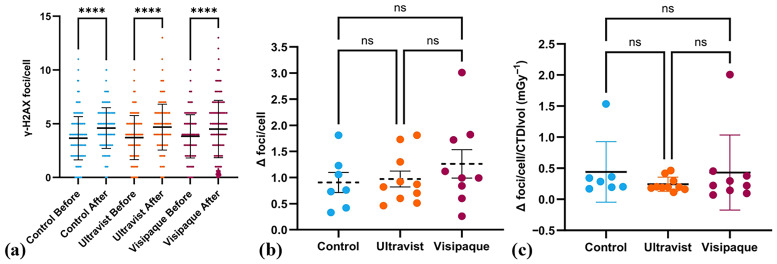
DNA damage response following pediatric chest CT. (**a**) Mean γ-H2AX foci per cell before and after CT for each group, shown as scatter plots with individual patient data points overlaid. All groups showed significant increases (*p* < 0.05, Wilcoxon signed-rank test). (**b**) Absolute change in γ-H2AX foci (Δ foci/cell) by group. Values represent mean ± SEM with individual patient data points overlaid. Despite numerical differences, between-group comparisons were not statistically significant (Kruskal–Wallis test, H = 1.092, *p* = 0.579, followed by Dunn’s multiple comparisons). (**c**) Dose-normalized change in γ-H2AX foci (Δ foci/cell/mGy) by group. No significant differences between groups (Kruskal–Wallis test, H = 0.834, *p* = 0.659). Statistical significance indicated as: ns, not significant; **** *p* < 0.0001.

Following the CT scans, there was a significant increase in the number of γ-H2AX foci for all three groups (*p* < 0.05 for each group using Wilcoxon signed-rank test, Figure 2a).

The patients who underwent unenhanced CT scans exhibited an increase of 0.915 ± 0.250 foci per cell, while patients who underwent Ultravist-enhanced CT scans showed an increase of 0.972 ± 0.279 foci per cell, and patients who underwent Visipaque-enhanced CT scans exhibited an increase of 1.261 ± 0.251 foci per cell (Figure 2b). This represented a 6.2% higher increase for Ultravist and 37.8% higher increase for Visipaque compared to the increase seen in patients who underwent unenhanced CT scans.

Despite the apparent numerical differences, statistical comparison using the Kruskal–Wallis test revealed no significant difference between the groups (H = 1.092, df = 2, *p* = 0.579). Post hoc analysis using Dunn’s multiple comparisons confirmed that none of the pairwise group comparisons reached statistical significance (all *p* > 0.05).

When DNA damage was normalized for radiation dose (CTDIvol) differences, the pattern changed substantially (Figure 2c). Dose-normalized changes were 0.344 ± 0.094 (unenhanced), 0.241 ± 0.069 (Ultravist), and 0.276 ± 0.055 (Visipaque) foci per cell per mGy, with no significant differences between groups (*p* = 0.659, Kruskal–Wallis test).

All γ-H2AX foci were validated through co-localization with 53BP1 foci, with >95% concordance confirming the specificity of the DNA damage assessment.

**Figure 3:**
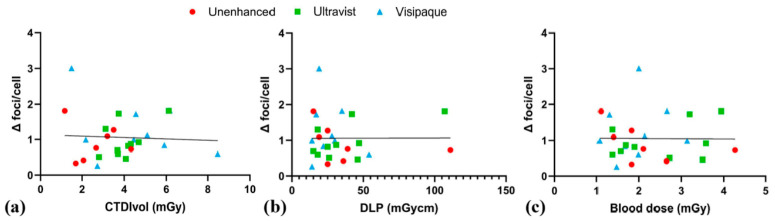
Dose–response relationships. (**a**) Change in γ-H2AX foci (Δ foci/cell) versus CTDIvol. (**b**) Change in γ-H2AX foci (Δ foci/cell) versus DLP. (**c**) Change in γ-H2AX foci (Δ foci/cell) versus NCICT-derived blood dose. Weak correlations were observed across all dose metrics, with substantial overlap between groups. Individual patient data points are shown by group: unenhanced (red circles), Ultravist (green squares), and Visipaque (blue triangles). Solid lines represent linear regression fits for each group.

Dose–response correlation analysis revealed weak correlations between γ-H2AX foci formation and various dose metrics (CTDIvol: Spearman’s ρ = 0.167, *p* = 0.414; DLP: ρ = −0.063, *p* = 0.762; blood dose: ρ = −0.043, *p* = 0.834) (Figure 3).

## 3. Discussion

### 3.1. Clinical Interpretation of Findings

Despite the higher administered radiation dose for contrast-enhanced examinations, whose increment ranged between 52 and 72% in CTDIvol (4.04–4.57 mGy vs. 2.66 mGy), and the apparently increased DNA damage for cases administered with contrast agents (6.2% for Ultravist, 37.8% for Visipaque), statistical analysis revealed no significant differences between groups in absolute DNA damage response (*p* = 0.579).

These findings contrast with several previous studies reporting significant enhancement effects of iodinated contrast agents on radiation-induced DNA damage. Piechowiak et al. (2015) [10] found 107% ± 19% higher γ-H2AX foci increases in contrast-enhanced chest CT without reporting dose equivalence between groups. Pathe et al. (2011) [23] observed 58% higher γ-H2AX foci levels in patients undergoing contrast-enhanced CT compared with unenhanced CT (*p* = 0.04); however, this difference was not significant at 1 h, 2 h, or 24 h post-CT (all *p* > 0.05). While Pathe tested for dose differences (DLP: 336 vs. 392 mGy·cm, *p* > 0.05), the small sample size (*n* = 15 per group) provided insufficient statistical power to confirm dose equivalence, with a 17% higher DLP in the contrast group remaining as a potential confounder. Wang et al. (2017) [24] reported 37.9% enhancement with dose-matched protocols (CTDIvol ~6.2 mGy, *p* = 0.978), suggesting biological enhancement may occur under controlled experimental conditions. These two approaches (dose-normalized clinical observation and dose-matched experimental design) address complementary but distinct questions: whether contrast produces disproportionate biological damage in routine clinical practice, and whether iodine is inherently radiosensitizing under controlled conditions, respectively. Our findings address the former, clinically relevant question. However, in routine clinical practice where contrast protocols employ substantially higher doses (our study: 52–72% higher CTDIvol), our dose-normalized analysis demonstrates no biological enhancement (*p* = 0.659). This distinction is important because while contrast agents increase physical radiation dose to tissues via photoelectric absorption [12], this does not translate to additional biological damage beyond that expected from dose alone at the cellular level in typical clinical practice. The 52–72% higher doses in our contrast protocols fully explain the 6.2–37.8% higher absolute DNA damage, with no additional biological effect.

No significant differences between groups were found even when DNA damage was adjusted for radiation dose differences (*p* = 0.659), demonstrating that contrast agents do not modify radiation-induced DNA damage response. While contrast-enhanced groups showed numerically lower damage per unit dose (Ultravist 30% less, Visipaque 20% less per mGy), these differences were not statistically significant and likely reflect expected statistical variation in small sample sizes rather than biological protection. Importantly, the absence of higher dose-normalized values in contrast groups contradicts the hypothesis of biological radiosensitization by iodinated contrast agents. This hypothesis, supported primarily by in vitro studies demonstrating dose enhancement ratios of 1.3–2.3 at clinically relevant iodine concentrations [8,18,25], does not appear to translate to in vivo conditions in pediatric patients. Our findings align with previous clinical studies. Beels et al. (2012) [11] demonstrated that γ-H2AX foci increase systematically with blood dose in patients, with in vitro experiments ruling out X-ray enhancement effects by contrast agents. Similarly, Gould et al. (2016) [20] found no evidence of contrast-mediated DNA damage enhancement in pediatric patients undergoing cardiac catheterization. Vandevoorde et al. (2015) [21] observed DNA damage in 51 pediatric patients undergoing chest and abdomen CT, demonstrating that γ-H2AX foci formation correlates with radiation dose and emphasizing the importance of dose optimization in pediatric populations. Van Cauteren et al. (2019) [26] investigated the effect of iodine dose on DNA damage in cardiac CT using a fixed scanner-reported CTDIvol protocol and found that higher iodine concentrations were associated with increased DNA damage. Their Monte Carlo model demonstrated that this increase was explained by corresponding increases in locally absorbed blood dose through the photoelectric effect (78.8% and 133.7% increases in locally absorbed blood dose for reduced and standard iodine protocols respectively), consistent with physical dose enhancement rather than biological radiosensitization. However, their model used a higher radiation dose than standard clinical cardiac CT protocols (CTDIvol 40.8 mGy, compared with 4.6–10.3 mGy reported in recent clinical coronary CT angiography studies) and a within-subject animal design, limiting direct comparison with clinical pediatric practice.

While recent Monte Carlo simulations have shown that contrast agents can increase organ radiation dose by an average of 30% due to photoelectric absorption enhancement [12], our biological data suggest that this physical dose increase does not translate into proportionally enhanced DNA damage at the cellular level. This dissociation between physical dose enhancement and biological effect may reflect complex factors including contrast agent distribution, cellular repair kinetics, and microdosimetric considerations that attenuate theoretical enhancement effects in vivo. The distinction between physical dose enhancement and chemical toxicity is critical for interpreting our results. In vitro studies at clinically relevant iodine concentrations demonstrate no significant enhancement of radiation-induced DNA damage by contrast agents [11], and baseline γ-H2AX foci levels in our study were similar across all groups (*p* > 0.05), confirming comparable pre-existing DNA damage levels and ruling out systematic differences in baseline radiosensitivity between groups. Our dose-normalized analysis accounts for physical dose increases due to photoelectric absorption while testing for additional biological effects beyond radiation alone. The absence of elevated dose-normalized DNA damage in contrast groups indicates that radiation dose, rather than contrast chemistry, is the primary determinant of DNA double-strand break formation under clinical conditions.

From a clinical perspective, these results indicate that contrast-enhanced CT poses no greater DNA damage risk than unenhanced CT when performed at equivalent radiation doses. This provides important reassurance for clinicians, particularly those treating pediatric patients where radiation sensitivity concerns are elevated. With allergic-like reactions to iodinated contrast being very low in pediatric populations (0.18–0.46%) [27,28], our findings suggest that DNA damage enhancement should not be an additional concern.

The risk–benefit calculation for contrast use should focus on diagnostic benefit versus baseline radiation risk, rather than concerns about enhanced radiation damage.

### 3.2. Study Limitations

Several important limitations must be acknowledged. The most significant is dose confounding, which prevented complete isolation of contrast agent effects from radiation dose effects. Clinical protocols necessitated different radiation doses for contrast versus non-contrast studies, as contrast-enhanced scans required higher doses to penetrate contrast-opacified tissues. This fundamental confounding prevents definitive isolation of pure contrast agent biological effects. However, our dose normalization approach and patient-specific Monte Carlo dosimetry provide the best available methods to address this limitation within ethical constraints in pediatric populations. Dose-matched protocols, while ideal for mechanistic studies, are only ethically feasible in adult volunteers or in vitro systems. Our clinically focused observational design with dose normalization provides evidence directly applicable to pediatric imaging practice, demonstrating that apparent contrast enhancement observed in previous clinical studies is primarily attributable to dose differences rather than biological radiosensitization.

Small sample sizes (7–10 patients per group) represent another significant limitation, restricting statistical power to detect small but potentially clinically meaningful differences. Post hoc power analysis suggests our study was adequately powered to detect only large effect sizes (Cohen’s d > 0.8), and the wide confidence intervals reflect this uncertainty. Larger multi-center studies would be needed to detect subtle contrast enhancement effects if they exist.

The non-randomized, observational design of this study introduces additional limitations regarding causal inference. Clinical indication determined treatment assignment, introducing potential selection bias if patients requiring contrast differ systematically in baseline DNA repair capacity or radiation sensitivity. While randomization to contrast versus no-contrast groups would be unethical when contrast is clinically indicated, the observational design limits definitive causal inferences, and residual confounding by indication remains possible despite statistical adjustments.

Additional methodological considerations warrant discussion when interpreting these results. The single timepoint assessment of DNA damage (30 min post-CT) was required by institutional review board restrictions on blood draw volumes in pediatric patients and represents an accepted standard based on kinetic studies showing peak γ-H2AX foci formation at 30–60 min post-irradiation at diagnostic doses [17]. While multiple timepoint sampling would provide more comprehensive kinetic data, such protocols were deemed ethically inappropriate given the additional burden to pediatric patients. The 30 min timepoint captures initial DNA damage formation, though it may not fully characterize repair kinetics. Future studies in adult populations or using less invasive sampling methods could address temporal dynamics of contrast effects on DNA damage and repair. Individual variability in contrast agent pharmacokinetics (renal function, hydration status, and clearance rates) represents another potential source of uncertainty that was not fully characterized in this study. While we employed multiple dosimetric calculations, each method carries inherent uncertainties related to anatomical modeling, age-specific conversion factors, and assumptions about blood distribution during scanning. The γ-H2AX foci assay, though well-established and sensitive, represents only one marker of DNA damage, and the correlation between blood dose and lymphocyte DNA damage may be influenced by factors such as cell cycle phase, individual radiosensitivity, and repair kinetics. The weak individual dose-damage correlations (Figure 3) are consistent with previous pediatric studies at diagnostic dose levels [19,21], where individual variability in radiosensitivity, repair capacity, and cell cycle distribution contributes to scatter at doses <10 mGy, and where dose–response relationships may follow non-linear low-dose hypersensitivity patterns rather than simple linearity [21]. While this limits our ability to perform precise individual risk estimates, it does not affect our primary conclusion: the absence of significant differences in dose-normalized DNA damage between groups demonstrates that contrast agents do not enhance biological damage beyond radiation dose effects. Despite careful blinding procedures, inter-observer variability in foci counting could influence results.

### 3.3. Future Research Directions

Future studies using dose-matched protocols could definitively address the dose confounding observed in clinical studies. Larger multi-center studies would provide more definitive evidence regarding subtle effects of contrast agents on DNA damage. Investigation of multiple timepoints post-exposure could reveal whether contrast agents influence DNA damage and repair kinetics, while additional biomarkers beyond γ-H2AX could provide a more comprehensive picture of cellular responses to contrast-enhanced radiation exposure.

## 4. Materials and Methods

### 4.1. Evaluation of DNA Double-Strand Breaks in Blood Samples Collected Before and After CT Scans in Pediatric Patients

#### 4.1.1. Subjects

The research included 26 children who underwent medically indicated chest CT scans at the radiology department of the UMC Heart Center (Astana, Kazakhstan) between March 2022 and November 2024.

The criteria for inclusion in the study were strict and included the following: (1) no previous exposure to X-rays; (2) absence of recent infections; (3) absence of major systemic diseases, blood-borne diseases, surgical history, or systemic medication use; and (4) no history of cancer or radiochemotherapy. Patients who did not meet all of these criteria were excluded from the study with the exception of one patient in the Ultravist group who had undergone CT examination ten months prior. While this met our minimum exclusion window, potential residual effects were considered in the analysis. For ethical reasons, patients requiring additional venipuncture solely for research purposes were excluded when catheter blood sampling was not feasible, particularly in very young patients. Based on clinical indication, patients were allocated to three groups: control patients receiving unenhanced CT scans, patients receiving Ultravist-enhanced CT scans, and patients receiving Visipaque-enhanced CT scans.

Before the CT examination, detailed information about each patient, including age, gender, height, weight, medical history, previous X-ray exposure, and exposure parameters, was recorded. Prior to blood collection, parental consent was obtained. The study received approval from the local ethical review boards of the participating hospital and of Nazarbayev University with the latter serving as the central oversight body. Written informed consent was obtained from all legal guardians.

#### 4.1.2. CT Equipment and Acquisition Protocol

The CT system used in the radiology department included Siemens SOMATOM Definition 64. The department had its own optimized pediatric CT protocol including low kV settings, automatic tube current modulation (Care Dose 4D), appropriate pitch values, and limited imaging lengths. Specific CT parameters for each chest CT scan are presented in Table 4.

During the CT scans, a tube voltage of 80 or 120 kVp was used, with the tube current automatically adjusting based on the patient’s needs, ranging from 18 to 150 mA. The imaging parameters included a rotation time of 0.5 s, table feed per rotation of 53.76 mm, detector width of 35.03 mm, scan field of view of 70 cm, and a reconstructed matrix of 512 × 512 pixels. The collimation of the CT scan was set to 64 × 0.6 mm, with a pitch of 1.4. For contrast-enhanced examinations, two non-ionic iodinated contrast agents were employed: Ultravist (iopromide, 370 mg I/mL, low-osmolar; Bayer AG, Berlin, Germany) and Visipaque (iodixanol, 320 mg I/mL, iso-osmolar; GE Healthcare, Chicago, IL, USA). Agent selection was based on clinical indication and patient characteristics following institutional protocols. Contrast was administered intravenously at weight-based dosing (1.2 mL/kg for Ultravist; 1.4 mL/kg for Visipaque) at an injection rate dependent on vascular access (1 mL/sec via 24G catheter; 1.5–2 mL/sec via 22G catheter). CT scanning was initiated automatically via bolus tracking when contrast reached the predetermined threshold, corresponding to early arterial phase, with an immediate second acquisition performed in the reverse direction without delay. The CTDIvol and DLP values reported represent the total accumulated radiation dose across all acquisitions including pre-monitoring, bolus tracking, and both post-injection scans.

#### 4.1.3. Blood Sample Collection, Lymphocyte Separation, Immunocytochemical Staining and Fluorescence Microscopy

The process of sample collection and isolation of lymphocytes involved obtaining two blood samples of 1 mL from each patient, one before and one 30 min after the CT exam, reflecting peak foci formation based on established kinetics [13]. Blood was collected in heparin-containing vials. The repair processes were halted by cooling the blood samples in ice. Blood was transported at 4 °C and processed. T-lymphocytes were isolated from the blood using a density gradient centrifugation technique. Briefly, the blood was diluted at a 1:1 ratio with phosphate-buffered saline (PBS), then slowly layered onto Histopaque 1077 (Sigma-Aldrich, St. Louis, MO, USA) to avoid any mixing and centrifuged at 1200× *g* for 20 min at room temperature (RT), and lymphocytes were washed twice in PBS. Resuspended T-lymphocytes (20 μL/well) were placed into 14 multi-well coated slides (Tekdon, Inc., Myakka City, FL, USA). The slides were then fixed with 2% formaldehyde (Sigma-Aldrich, St. Louis, MO, USA) for 10 min and stored overnight in 0.5% formaldehyde. The following day, the samples were permeabilized for 5 min by adding 100 μL of 0.25% Triton-X-100 (Sigma-Aldrich, St. Louis, MO, USA)/well and then blocked in PBS with 1% bovine serum albumin for 10 min at RT. The samples were incubated for 45 min with mouse anti-γ-H2AX antibody (clone 2E3, 1:500; BioLegend, San Diego, CA, USA) and rabbit anti-53BP1 antibody (1:500; Abcam, Cambridge, UK), washed in PBS and 1%BSA three times for 5 min each, and incubated with Alexa Fluor 488-conjugated goat anti-mouse (1:500; Life Technologies, Paisley, UK) and Alexa Fluor 555-conjugated goat anti-rabbit secondary antibodies (1:500; Invitrogen, Paisley, UK) for 45 min at room temperature. The cells were then washed in PBS three times for 5 min each and mounted by using ProLong Gold Antifade Mountant with 4′,6-diamidino-2-phenylindole (DAPI) (Thermo Fisher Scientific, Waltham, MA, USA).

Fluorescence images were acquired by using a Leica Thunder Imager microscope (Leica Microsystems, Wetzlar, Germany) equipped with Leica DFC9000 GTC VSC-13069 camera and acquisition software (LAS X) version 5.3.1. For quantitative analysis, the foci were counted at 100× objective magnification. Monocytes and granulocytes were distinguished using morphological characteristics when viewed under the microscope and were subsequently excluded from the analysis. As a result, the data reflects a lymphocyte-like morphology population. For each patient, approximately 100 lymphocytes were analyzed to determine the mean number of γ-H2AX foci per cell. The slides were coded and the quantification of focus numbers per cell was carried out by an observer who was unaware of the sample’s treatment type (control, Ultravist-enhanced CT, Visipaque-enhanced CT, before or after CT). The CT-induced γ-H2AX foci yield for each patient was calculated by subtracting the pre-scan mean foci count from the post-scan mean foci count.

**Figure 4:**
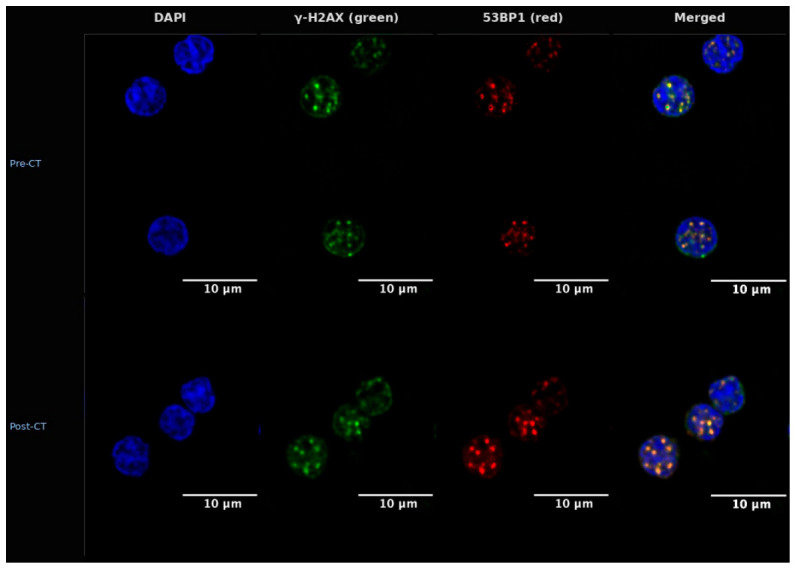
Representative high-magnification fluorescence microscopy images of γ-H2AX and 53BP1 foci in peripheral blood T-lymphocytes. Upper panel: Pre-CT baseline sample. Lower panel: Post-CT sample (patient 8, Ultravist, 30 min post-examination). Columns from left to right: DAPI nuclear stain (blue); γ-H2AX immunofluorescence (Alexa Fluor 488, green); 53BP1 immunofluorescence (Alexa Fluor 555, red); merged image. Co-localized foci appear as yellow/orange in the merged image, representing spatial overlap of γ-H2AX and 53BP1 signals and indicating genuine DNA double-strand break sites. Only co-localized foci were scored as positive DSBs. Scale bars: 10 μm. ×100 objective magnification, Leica Thunder Imager fluorescence microscope.

DNA double-strand breaks were quantified by scoring γ-H2AX foci co-localized with 53BP1 foci. This dual-marker approach ensures specificity by discriminating genuine DSBs from background artifacts. At the 30 min post-CT timepoint, both γ-H2AX and 53BP1 are robustly present at DSB sites. Schultz et al. (2000) [29] demonstrated that 53BP1 foci peak at 30 min post-irradiation and show remarkable co-localization with γ-H2AX foci at this timepoint, confirming that 30 min represents an appropriate sampling window for dual-marker DSB quantification. Only foci showing overlapping signals for both markers in merged fluorescence images were counted as positive (Figure 4). Each patient’s result represents the mean response from their individual lymphocyte population, with these patient-level means serving as the independent data points for subsequent statistical analysis.

### 4.2. Patient Dosimetry

Organ dose and effective dose (ED) were calculated by using the National Cancer Institute dosimetry system for computed tomography (NCICT) [22] which employs Monte Carlo simulations with age-specific computational phantoms. For the computation, we used age-specific conversion factors selecting the age closest to that of the specific patient. NCICT provides discrete age-specific phantoms (newborn, 1-year, 5-year, 10-year, 15-year). Patients were assigned to phantoms using nearest-age matching: 0–2 years to the 1-year phantom (*n* = 18); 3–7 years to the 5-year phantom (*n* = 6); and 8–13 years to the 10-year phantom (*n* = 2).

For each patient, we input scanner model Siemens SOMATOM Definition 64 (Siemens Healthineers, Erlangen, Germany), patient age, measured CTDIvol values, and technical parameters (pitch 1.4, tube voltage 80 kV or 120 kV). CTDIvol values were adjusted within the software to match recorded DLP values. The software calculated organ-specific doses for lung, heart wall, and bone marrow. We focused particularly on these due to their anatomical location within the scan field. Blood dose was derived from NCICT organ doses by weighting each organ dose by its blood volume fraction from ICRP Publication 89 [30], following the methodology in refs. [11,31,32]:

(1) $$Blood\;dose=\frac{\left[(0.16\;\times \;Heart\;wall\;dose)\;+\;(0.125\;\times \;Lung\;dose)\;+\;(0.07\;\times \;Active\;marrow\;dose)\right]}{0.355}$$

where the coefficients represent the fraction of total blood volume distributed in cardiac chambers (16%), pulmonary circulation (12.5%), and bone marrow (7%), and the denominator normalizes for the 35.5% of total blood volume captured in these three organs within the chest scan field.

For correlation analyses with γ-H2AX/53BP1 foci formation, we used blood dose estimates from the NCICT organ dose estimates.

Correlations between DNA damage biomarkers (γ-H2AX/53BP1 foci) and dosimetric parameters (CTDIvol, DLP, and estimated blood dose) were evaluated using Spearman’s rank correlation coefficients to account for potential non-linear relationships. Dosimetric analysis employed complementary approaches: (1) scanner-reported CTDIvol and DLP from DICOM headers, (2) patient-specific blood doses calculated using NCICT Monte Carlo simulation accounting for individual anatomy and scan coverage, and (3) normalization of DNA damage by CTDIvol to enable comparison across different scanning protocols. This multi-metric approach ensures that conclusions are robust to differences in scan parameters including anatomical coverage.

### 4.3. Statistical Analysis

Data are presented as mean ± standard error of the mean (SEM). Each patient was treated as an independent experimental unit (*n* = 7 Control, *n* = 10 Ultravist, *n* = 9 Visipaque patients). Statistical comparisons were performed using the Wilcoxon signed-rank test for paired comparisons within each group (before vs. after CT) and the Kruskal–Wallis test followed by Dunn’s multiple comparisons test for comparing change scores between the three treatment groups. Non-parametric tests were chosen due to the small sample sizes and to avoid assumptions about data distribution. A *p*-value of <0.05 was considered statistically significant. All statistical analyses were performed using GraphPad Prism version 10 (GraphPad Software, Inc., San Diego, CA, USA).

## 5. Conclusions

This study provides reassuring evidence that iodinated contrast agents do not enhance radiation-induced DNA damage beyond what would be expected from radiation dose alone in pediatric patients undergoing chest CT. These results specifically address routine clinical practice where contrast protocols require higher radiation doses and do not exclude the possibility of biological radiosensitization under perfectly dose-matched experimental conditions. The data support the safety of current clinical practices regarding contrast use and suggest that radiation enhancement concerns should not influence clinical decision-making about contrast administration. This is particularly meaningful in pediatric radiology, where radiation sensitivity is a well-founded concern, and provides important evidence supporting the continued appropriate use of contrast-enhanced CT when clinically indicated.

## Figures and Tables

**Table 1:** Demographic data of the patients included in the study.

	Chest CT
Age (months)	Average 24.03 (range 1.8–123)
Weight (kg)	Average 9.97 (range 4.2–28)
Height (cm)	Average 80.42 (range 55–144)
Males	15
Females	11

**Table 2:** Patient characteristics and dose parameters by group *.

Patients Characteristic	Unenhanced CT (*n* = 7)	Ultravist-Enhanced CT (*n* = 10)	Visipaque-Enhanced CT (*n* = 9)
Male	5 (71.4%)	6 (60%)	4 (44.4%)
Female	2 (28.6%)	4 (40%)	5 (55.6%)
Age (months)	28.4 ± 12.7	22.2 ± 11.8	22.6 ± 12.6
Age- Male (months)	19.0 ± 5.6	13.0 ± 6.0	11.4 ± 4.0
Age- Female (months)	65.5 ± 30.5	36.0 ± 27.6	31.4 ± 22.5
Height (cm)	84.3 ± 7.9	81.7 ± 8.5	80.0 ± 8.6
Weight (kg)	10.7 ± 2.3	9.6 ± 2.3	9.5 ± 1.8
Volumetric CT dose index (mGy)	2.66 ± 0.42	4.04 ± 0.31	4.57 ± 0.76
Dose-length product (mGy·cm)	38.6 ± 14.0	37.5 ± 8.7	25.9 ± 4.7
Scan length (cm)	14.02 ± 2.54	9.58 ± 0.88	6.78 ± 1.12

* Data are mean ± SEM. Scan length (cm) = DLP (mGy.cm)/CTDIvol (mGy).

**Table 3:** NCICT-calculated organ doses by group *.

Parameter	Unenhanced (*n* = 7)	Ultravist (*n* = 10)	Visipaque (*n* = 9)
Effective dose (mSv)	2.00 ± 0.35	2.20 ± 0.26	1.93 ± 0.22
Lung dose (mGy)	4.66 ± 0.91	5.37 ± 0.69	4.17 ± 0.50
Heart wall dose (mGy)	4.70 ± 0.92	5.36 ± 0.69	4.19 ± 0.50
Active marrow dose (mGy)	1.30 ± 0.19	1.40 ± 0.19	1.17 ± 0.15
Blood dose (mGy)	2.17 ± 0.42	2.49 ± 0.32	1.94 ± 0.23

* Data presented as mean ± SEM.

**Table 4:** Individual CT parameters for all patients undergoing chest CT examination.

Patient Number	Tube Current (mA)	Exposure Time (s)	Tube Voltage (kV)	Pitch	Collimation (mm)	Current-Exposure Time Product (mAs)	CTDIvol (mGy)	DLP (mGy.cm)	Contrast Agent Given
1 *	113.5	0.5	80 kV	1.4	38.4	1959	4.69	47	Ultravist
2	150	0.5	80 kV	1.4	38.4	4039	6.12	107	Ultravist
3	71	0.5	80 kV	1.4	38.4	1166	2.81	26	Ultravist
4	95	0.5	80 kV	1.4	38.4	1912	4.08	46	Ultravist
5	84.5	0.5	80 kV	1.4	38.4	1711	3.74	42	Ultravist
6	61.5	0.5	80 kV	1.4	38.4	1886	8.47	54	Visipaque
7	**	0.5	80 kV	1.4	38.4	1153	2.18	30	Visipaque
8	40	0.5	80 kV	1.4	38.4	992	4.19	25	Ultravist
9	35	0.5	120 kV	1.4	38.4	1117	4.33	111	Unenhanced
10	18	0.5	120 kV	1.4	38.4	190	1.18	15	Unenhanced
11	**	0.5	80 kV	1.4	38.4	712	1.5	19	Visipaque
12	26	0.5	120 kV	1.4	38.4	292	1.7	25	Unenhanced
13	24.5	0.5	80 kV	1.4	38.4	535	2.73	14	Visipaque
14	43.5	0.5	80 kV	1.4	38.4	1230	6.17	35	Visipaque
15	35.5	0.5	80 kV	1.4	38.4	754	5.91	22	Visipaque
16	33	0.5	120 kV	1.4	38.4	406	2.07	36	Unenhanced
17	25.5	0.5	80 kV	1.4	38.4	626	3.7	18	Ultravist
18	35.5	0.5	80 kV	1.4	38.4	682	3.12	18	Ultravist
19	39.5	0.5	80 kV	1.4	38.4	988	5.1	28	Visipaque
20	44	0.5	80 kV	1.4	38.4	1087	4.32	31	Ultravist
21	24	0.5	80 kV	1.4	38.4	509	3.68	15	Ultravist
22	43	0.5	120 kV	1.4	38.4	414	2.67	39	Unenhanced
23	47	0.5	80 kV	1.4	38.4	670	4.56	17	Visipaque
24	41	0.5	80 kV	1.4	38.4	512	4.46	14	Visipaque
25	27	0.5	120 kV	1.4	38.4	302	3.5	25	Unenhanced
26	25	0.5	120 kV	1.4	38.4	280	3.2	19	Unenhanced

* Note: Patient 1 had a previous scan 10 months prior to our study enrolment. ** Tube current values unavailable for patients 7 and 11.

## Data Availability

Anonymized summary data are contained within the article. Individual patient-level data are available on request from the corresponding author, subject to institutional ethics committee approval and data privacy regulations.
